# Comparing the Impact of Nature, Blended, and Traditional Preschools on Children’s Resilience: Some Nature May Be Better Than None

**DOI:** 10.3389/fpsyg.2021.724340

**Published:** 2021-10-13

**Authors:** Julie Ernst, Hannah Juckett, David Sobel

**Affiliations:** ^1^Applied Human Sciences, University of Minnesota Duluth, Duluth, MN, United States; ^2^Environmental Education, University of Minnesota Duluth, Duluth, MN, United States; ^3^Education, Antioch University New England, Keene, NH, United States

**Keywords:** nature preschools, resilience (psychological), protective factors, initiative, self-regulation, attachment

## Abstract

This study examined the effect of nature preschools on the development of key protective factors associated with psychological resilience. The Deveraux Early Childhood Assessment for Preschoolers, Second Edition (DECA-P2), was used to assess the growth in the protective factors of initiative, self-regulation, and attachment in 87 children who attended nature, blended, and traditional preschool classes within the same school district. Study results suggest that nature preschool participation was important in the context of initiative. Blended classes, where some nature-based practices were incorporated into traditional preschool classes, were sufficient in the sense of being more impactful than traditional classes on self-regulation, attachment, and the total protective factors overall. Implications are discussed within the context of the limitations of the study.

## Introduction

In recent years, concerns regarding declining resilience in children have surfaced in the academic and popular literature, alongside concerns regarding increasing stress, anxiety, and depression (Grey, 2013; Masten and Barnes, 2018). Resilience is a particularly relevant psychological construct to explore, especially in light of the coronavirus pandemic. Children have experienced significant stress throughout the pandemic, including quarantining at home, increased screen time, limited access to extended family members and playmates, and the anxiety of caregivers regarding getting sick (American Academy of Pediatrics, 2021). And for some children, the pandemic resulted in stress from financial hardships, fear from increased tensions in households, and grief from the loss of loved ones (Bartlett et al., 2020). While children generally and eventually return to their typical functioning, particularly with responsive and supportive caregivers, some are at risk of developing trauma-related stress, anxiety, and depression (Bartlett et al., 2020).

Psychological resilience is commonly described as the ability to recover from adversity, whether severe and prolonged adversity, such as the pandemic, or on a smaller scale, such as the difficulties that surface as a part of daily life. Resilience is malleable. It is not a characteristic that children either have or do not have; yet, differences in children’s personalities and cognitive skills influence adaptive capacity, as do their connections to other people and to external systems (Masten and Barnes, 2018). Benard (2004) defines resilience as being able to overcome adversity and become competent and caring individuals, and Luthar et al. (2000) positions resilience as the positive, adaptive response in the presence of adversity. With the relatively recent ability to study resilience at the neurobiological level, Masten (2014) describes resilience as embedded within interacting processes and systems, including molecular-level systems within the individual (including epigenetic and immune system processes), social-level systems (families, friends, etc.), community-level systems (schools, emergency service systems, etc.), and macrosystems (such as government-level systems that indirectly influence other systems through policies and regulations). These interacting processes and systems shape the course of children’s development and adaptation (Masten, 2014). Consequently, Masten and Cicchetti (2016) conceptualize resilience as the capacity of a dynamic system to adapt to disturbances that threaten the development of the system or its function or viability. For an individual, “resilience reflects all the adaptive capacity available at a given time in a given context that can be drawn upon to respond to current or future challenges facing the individual, through many different processes and connections” (Masten and Barnes, 2018).

Over time, research on resilience has shifted from identifying risk factors to studying what enables children to thrive in spite of adversity, thus transitioning from a problem-based deficit model to a strengths-based model (Masten, 2007). There are protective mechanisms that support successful adaptation to adversity (Benard, 2004). These within-child dispositions and skills, such as problem-solving abilities, initiative, a sense of self-efficacy, self-regulation skills, persistence, and a sense of purpose and belief that life has meaning (Wright and Masten, 2005). Protective factors can involve supports, such as positive relationships with caring and competent adults, effective parenting, positive friendships, effective schools and teachers, and protecting and nurturing brain development (Masten et al., 2008); these have been described as harnessing or restoring the power of human adaptive systems (Masten, 2014).

Somewhat missing, however, in the extensive body of literature regarding protective mechanisms is a solid recognition of the importance of nature exposure and/or positive human connections with the natural world (Wells, 2013; Chawla et al., 2014). This lack of prominent recognition exists in spite of a growing number of studies that connect nature to resilience-related outcomes. For example, Chawla et al. (2014) investigated green schoolyards’ impact on children’s stress and resilience. The findings from their ethnographic study suggest children experienced not only restoration in their green schoolyards, but also developed feelings of competency and developed supportive social relationships, both of which are considered protective factors relating to resiliency (Chawla et al., 2014). McArdle et al. (2013) investigated the effect of a preschool program that used outdoor free play and intentional efforts to provide nurturing relationships for children who had experienced disruptions in attachments early in life. Their ethnographic study suggests a strengthening of confidence in the face of new challenges, self-control, empathy, motivation, focus, and perseverance (McArdle et al., 2013). A quantitative study by Ernst et al. (2018) found that students enrolled in a nature preschool showed significant strengthening of their total protective factors related to resilience over the course of one school year. Results from Ritchie et al. (2014) suggest the potential for multi-day outdoor adventure trips to influence the resilience and well-being of adolescents, and Buchecker and Degenhardt (2015) found a positive (but modest) relationship between outdoor recreation in nearby nature and urban adults’ emotional well-being and resilience. Additionally, there has been some study of single protective factors such as the influence of nature exposure or experiences on self-regulation (Fabor Taylor et al., 2002; McCree et al., 2018; Weeland et al., 2019).

Empirical evidence and theory provide two plausible linkages, or mediating mechanisms, between nature exposure and resilience (Wells, 2013). One of these is that access to nature fosters social interactions and supports the development of social relationships. The potential for green settings to draw people together, thereby foster social interactions, friendships, and social ties is evident in several studies (e.g., Kuo et al., 1988; Coley et al., 1997; Fabor Taylor et al., 1998; Sullivan et al., 2004). The second is that access to nature boosts cognitive functioning. This is grounded in attention restoration theory (Kaplan and Kaplan, 1989), which suggests natural environments can counter directed attention fatigue, as nature engages involuntary attention and thus allows directed attention capacities to recharge. Studies, such as Wells (2000), Fabor Taylor et al. (2001), and Berto (2005), support this association between nature exposure and successful attentional capacity and day-to-day cognitive functioning, both of which help with management of adversity. Social interactions and cognitive functioning are strong predictors of resilience (Masten, 2007), thereby providing the link between nature and resilience.

While these mediating mechanisms proposed by Wells (2013) are in the context of nature and not specifically nature preschool, they provide foundational support for the hypothesized association between nature preschool and resilience. Additionally, in light of research that connects nature preschools to positive social relationships and cognitive functioning (e.g., Cordiano et al., 2019; Ernst and Burcak, 2019; Volpe et al., 2019; Bal and Kaya, 2020; Robinson and Ernst, 2020), it seems reasonable to further study the relationship between nature preschools specifically and resilience. Additionally, further research focusing on young children in the context of resilience is needed. Masten et al. (2008) notes that even though resilience can be supported at every age, there are certain windows of opportunity where supporting the development of protective factors and harnessing the power of protective factors and systems are especially important. One of those critical windows is early childhood and heightened brain plasticity (Center on the Developing Child at Harvard University, n.d.). Additionally, Masten et al. (2008, p. 79) indicate “competence begets competence,” and thus, investing early is recommended, and there is a high return on investment in early child development documented by Heckman (2006). Consequently, the study at hand sought to further explore the potential of nature preschools to support the development of protective factors associated with resilience in young children.

## Materials and Methods

### Purpose

The purpose of this study was to investigate whether nature preschool fosters the growth of young children’s total protective factors associated with resilience, and how that growth compares with preschool classes where there is less incorporation of nature-based approaches and experiences. The specific research question guiding the study was as follows: When controlling for preschool participants’ pretest levels, gender, and dosage of participation, do nature preschoolers have higher levels of protective factors (operationalized as initiative, self-regulation, and attachment) at the end of the school year than their peers in blended and non-nature preschool classes?

### Participants

The participants in this study were preschool-aged children enrolled in public preschool classes within one school district, located in a Midwest (United States) suburban area just outside of a major metropolitan area. The school district enrolls approximately 9,000 students from across several cities and townships. The median household income in this community is roughly $100,000, and the community is approximately 90% White (National Center for Education Statistics, 2020). This public preschool system was chosen because of the unique scenario of multiple classes of nature, blended, and traditional preschool programming all within the same school district.

All 17 preschool classes were invited to participate in this study. While initially all teachers expressed interested, as the start of the school year approached and with the uncertainty of the coronavirus pandemic and what the school year would bring, some decided not to participate, resulting in 11 classes who participated in the study. These 11 classes were taught by six teachers, as several teachers had two or three classes, such as a Monday–Wednesday–Friday morning class and a Tuesday–Thursday morning class. Following school district and University Institutional Review Board Approval, all preschoolers in the 11 classes were invited to participate in the study, with the exception of children who were receiving special education and spent the majority of their preschool day not in the preschool classroom. Six teachers taught the 11 participating classes, and there were a total of 87 children with parental consent to participate (see Table 1).

**Table 1:** Summary of class and participant data.

Category	Teacher/class	Degree of “Nature-ness”^a^	# of Students	Average Age (mos.)^b^	% Female	Weekly Attendance	Days distance learning due to COVID^c^
Traditional	1/a	22	8	45	63	5 half days	20
	1/b	22	8	55	63	5 half days	20
	2/a	29	7	63	43	5 full days	0
	3/a	29.5	5	58	60	5 half days	0
	3/b	29.5	9	60	44	5 half days	0
Blended	4/a	33	5	42	60	3 half days	0
	4/b	33	8	41	75	2 half days	0
	4/c	33	9	47	44	3 half days	0
Nature	5/a	36.5	8	44	38	3 half days	4
	5/b	36.5	7	54	57	2 half days	5
	6/a	36.5	13	56	54	5 full days	21

a *Score on Nature-ness Rubric, ranging from 13 to 39, with higher scores indicating higher levels/more nature-based settings, practices*, etc.

b *Age at time of the pretest*.

c *All classes had distance learning November 23–34, November 30–December 4; days indicated in column are in additional to program-wide distance learning*.

### Design

A quasi-experimental design (non-equivalent pretest-post-test design) was used in this study. The district offers three types of preschool programming: nature, blended, and traditional. In addition, classes vary by duration (“dosage”), with some being half-day and others being full day; some classes occur 2 or 3days per week, and others occur 5days per week. Parents select the type of preschool and the specific class when they enroll their preschoolers, and thus, random assignment was not feasible.

The nature classes were considered the treatment group; the blended and traditional classrooms served as comparison groups. Nature preschool classes were located at two sites (an elementary school and the early learning center) and focused on developing curiosity, a love of learning, and a respect for/connection to nature through playful, unstructured or loosely guided experiences in and with nature. In the nature preschool classes, teachers primarily used child-directed activities, based on the interests of their students and what emerged from their interactions in nature. These classes spent most of their time outdoors in an unmaintained natural setting (3–5h, depending on half or full day class sections). The nature preschool classes at the elementary site had access to 80 acres of city land designated for wildlife conservation and environmental education purposes. This setting offers several ponds, a stream, prairie areas, forest areas, wetlands, and multiple walking paths. There is also a nature playscape at this site, consisting of a mud kitchen, woodblocks, tree stumps, and other features typical to nature playscapes. The nature preschool classes at the early learning center used a variety of natural space around the perimeter of the school, which allowed for play and exploration in both wooded and grassy areas. The playscape at this site had a mud kitchen, fort-building/sticks, and other natural loose parts (tree cookies, rocks, etc.). The area is about one-quarter acre in size and much of it is a hilly slope covered with sumac and other emergent vegetation just a bit taller than the children. At the base of the hill, there is a paved path, as well as a stump circle for gathering, and a compost bin. There is also a drainage ditch that periodically fills with water and/or ice.

Traditional preschool classes focused on developing early literacy and math skills, through a combination of teacher-directed instruction and child-directed play to prepare children for Kindergarten. The traditional preschool classes were located at the early learning center. Play occurred primarily indoors, with about a half hour of weather-dependent outdoor play (typically on the nearby elementary school playground with occasional use of the nature playscape), as well as use of an outdoor courtyard with riding toys, wagons, plastic trucks, toy figures, etc. While nature was not a focus of the traditional classroom, the children had opportunities to learn about nature, such as using apples or pumpkins in the fall to practice counting and letters and in the context of science (learning about what plants need to grow), for example. Thus, nature was included as subject matter, as opposed to serving as an immersive setting for child-directed play as in the nature preschool classes.

The blended preschool classes were also located at the early learning center. Similar to the traditional classes, the blended classes were focused on Kindergarten readiness, but also had an aim of connecting children to nature. Blended classes balanced teacher-directed instruction and child-directed play, similar to the traditional classes, but also included about an hour of outdoor play in nature and/or teacher-guided outdoor learning. Although the playground and courtyard were used for outdoor play, there was regular use of the nature playscape as well.

While participating preschool classes were already classified as either nature, blended, or traditional, the assignment of teachers to specific classes opened the possibility for a blurring of lines between the categories (e.g., a teacher with a more traditional preschool teaching philosophy or experience base being assigned to a blended class). For this reason and to confirm the labeling (categorizing) of classes into the treatment and comparison groups, a rubric was developed, building on the work of Bailie (2016) and Larimore et al. (2019), and field tested prior to use in the study at hand (see Table 2). The rubric has a total of 13 items covering four traits: curriculum and instructional practices, nature-related curriculum and instruction practices, teacher role, and indoor and outdoor environments. In terms of scoring, for the rows with three cells, points were awarded as follows: three points for the left cell, two points for the middle cell, and one point for the right cell. For rows with two cells, the cell on the left was three points and the cell on the right was one point. If teachers circled two cells, then the points were the average of the two cells, for example, 2.5 if they circled both the left and middle cells in a row. Higher total points indicate higher degree of “nature-ness,” with the highest possible level on this rubric being 39 and the lowest being 13.

**Table 2:** Preschool “nature-ness” categorization rubric.

	Nature	Blended	Traditional
1.	Instructional focus is on both environmental outcomes (nature connection, sense of placsse, respect for nature) and Kindergarten preparation; Kindergarten prep focuses on developing curiosity, love of learning, problem-solving, independence, as well as other social–emotional outcomes	Instructional focus is on Kindergarten preparation, including developing early literacy and math skills and fostering positive in-classroom behaviors, as well as other social–emotional outcomes	
2.	Social and Emotional Learning (as well as other desired outcomes) accomplished primarily through nature play and/or playful, guided outdoor learning, as well as teacher-guided negotiations.	Social and Emotional Learning (as well as other desired outcomes) accomplished through a combination of developmentally appropriate direct instruction, curriculum materials, indoor play, outdoor play, and outdoor play in nature	Social and Emotional Learning (as well as other desired outcomes) accomplished through developmentally appropriate direct instruction and curriculum materials, as well as through play (primarily indoors)
3.	Majority of the day is not teacher-directed	Relatively equal use of teacher-directed activities and child-directed activity	More of the day is teacher-directed than child-directed
4.	Classroom management toward positive behaviors, emphasizes developing empathy and community	Classroom management toward positive behaviors involves a combination of classroom expectations, classroom rules, and developing empathy and community	Classroom management approach oriented toward classroom expectations and rules
5.	Substantial focus on child-directed nature play	Some child-directed nature play encouraged	A small amount of child-directed nature play encouraged
6.	Some teacher-guided nature learning outdoors (with a greater emphasis on child-directed playful learning when outdoors)	Some teacher-guided learning outdoors	Small amount of teacher-guided learning outdoors
7.	Much impromptu nature learning based on what’s found outdoors/in nature (including weather-related)	Some impromptu nature learning based on what’s found outdoors/in nature (including weather-related)	Infrequent impromptu nature learning outdoors
8.	During outdoor playtime, teacher joins in play, helps set the stage for play, models play skills or behaviors, and/or observes play toward understanding children’s interests and play habits	During outdoor play, teacher primarily observes and/or actively guides play, toward maintaining safety and appropriate child behavior and interactions	
9.	Time in the indoor classroom is primarily child-driven. The teacher sets up the indoor classroom with open-ended activities for children to choose from	Inside, teachers lead small and/or large group activities along with providing time for child-directed play	Inside, teachers structure, organize, and often lead activities for children. There is an emphasis on teacher-designed activities for children
			10.	Emphasis on respect for nature and others (equal emphasis)	Emphasis on respect for nature and others, with slightly more emphasis on respect for others	Emphasis on a respect for others (and a respect for nature as secondary)
11.	Teachers allow children to work out conflicts on their own as much as possible.	Teachers balance child and teacher negotiation strategies to resolve conflicts.	Teachers provide guided negotiation when conflicts arise.
12.	Indoor environment includes substantial nature content in wall displays, classroom materials, etc. Classrooms softly lit	Indoor environment has some nature content.Classrooms brightly lit	Indoor environment emphasizes other things relevant and of interest to preschoolers. Classrooms brightly lit
		13.		Outdoor environment used is primarily an unmaintained, natural setting(s); a maintained natural playspace is also available	Variety of outdoor environments used, including unmaintained natural area, maintained naturalized outdoor play space, and outdoor playground	Outdoor environment used is primarily outdoor playground, with a naturalized outdoor play space and natural environment also available

*Rubric builds upon the work of* *Bailie (2016)* *and* *Larimore et al. (2019)*.

The six teachers completed this rubric near the end of the academic year, and their “nature-ness” scores were used to confirm the categorization of their classes (See Table 2). Based on the completed rubrics, the two teachers with the level of “nature-ness” had a “score” of 36.5; these two teachers taught classes that were labeled by the district as nature preschool classes. The teacher of the three sections of district’s blended classes had a “nature-ness” score that was lower than the teachers of the nature sections (a score of 33), and the three teachers of the classes labeled by the district as traditional had the lowest “nature-ness” scores (scores of 29.5, 29, and 22). Thus, while scoring confirmed the general categorization into groups, it is important to note that “nature-ness” or degree of nature-based preschool was more of a continuum, rather than discrete categories. Even the classes in the traditional category had some degree of “nature-ness,” as indicated by their rubric scores, likely due to the district being an E-STEM (Environmental, Science, Technology, Engineering and Math) district.

### Construct and Measure

The Devereux Early Childhood Assessment for Preschoolers, Second Edition (DECA-P2) (LeBuffe and Naglieri, 2012), was used in this study to measure within-child protective factors central to resilience and social–emotional well-being. This behavior rating scale is completed by teachers and/or parents and evaluates the frequency of 27 positive behaviors (strengths) exhibited by preschoolers during the prior 4weeks, on a five-point scale from never to very frequently. This instrument includes instructions for those completing the rating form and is designed to be used without specific training. Due to the pandemic and in efforts to not add further stress to parents and families, parents were not asked to complete the DECA-P2 for their children; only the teachers were asked to do so.

The DECA-P2 has three subscales assessing within-child protective factors: initiative, self-regulation, and attachment/relationships, which are described in more detail in the manual (LeBuffe and Naglieri, 2012). The *initiative* subscale contains nine items measuring the child’s ability to use independent thought and action to meet his or her needs. Example items within this subscale are “show an interest in learning new things” and “make decisions for him/herself.” The *self-regulation* subscale contains nine items that measure the child’s ability to express emotions and successfully manage behaviors. Example items include “handle frustration well” and “accept another choice when his/her first choice was not available.” The *attachment* subscale contains nine items that measure the child’s ability to promote and maintain mutual, positive relationships or connections with other children and adults. Example items include “trust familiar adults and believe what they say” and “seek help from others when necessary.” A child’s score for each of the subscales is calculated by summing the scores of the nine items within the subscale.

The DECA-P2 assessment has been demonstrated to be reliable and valid; the specific *total protective factors* score has also been described as “the most reliable and valid overall indicator of strengths related to resiliency” relative to its three subscales (LeBuffe and Naglieri, 2012, p. 92). The reported internal reliability coefficient for the overall total protective factors scale is 0.95 for teachers. For the subscales, the initiative reliability coefficient is 0.92, self-regulation is 0.94, and relationship is 0.85 (LeBuffe and Naglieri, 2012). Content validity was established during development of the test, using a combination of focus groups and literature reviews on social and emotional competence and resilience in young children (LeBuffe and Naglieri, 2012). Criterion validity was established using comparisons across different samples to measure the degree to which the scores on the assessment predict an individual’s performance on an outcome (LeBuffe and Naglieri, 2012). Construct validity was established by correlating *T*-scores on the DECA-P2 with standard scores from the Preschool Behavioral and Emotional Rating Scale and the Conners Early Childhood Scale (for more information, see LeBuffe and Naglieri, 2012).

### Data Collection Procedures

Teachers were asked to complete the DECA-P2 for each child for whom parental consent had been granted. Teachers were also asked to complete a coding sheet, where children’s assessment forms were labeled with a code rather than a child’s name, to ensure data confidentiality while allowing for linking the pretest and post-test data. In addition, the following demographic data were collected through the coding system: children’s age, gender, and “dosage” of preschool (full day/half-day and days/week). Demographic data regarding socio-economic status, race, and ethnicity were not collected, due to the lack of variation and to avoid being able to identify specific participants. Teachers completed the DECA-P2 on two occasions, at the beginning of the school year (4weeks into the school year, per DECA-P2 instructions, which suggest a four-week period of getting to know the children prior to completing the assessment), and again at the end of the academic year.

The pre- and post-assessments were scored according to the scoring procedure in DECA User’s Guide and Technical Manual (LeBuffe and Naglieri, 2012). The raw scores for the overall total protective factors and three subscales were converted to standard scores (T-scores), using tables provided in the manual. According to LeBuffe and Naglieri (2012), T-scores are classified as a protective factor “strength” (T-score of 60–72), “typical” (T-score of 41–59), or “area of need” (total protective factor T-score of 28–40). As directed by the manual, the T-scores were used in pretest-post-test comparisons at the child- and/or program-levels.

### Analytic Strategy

Descriptive statistics were used to compute and summarize the means and standard deviations of the pretest and post-test scores for the total protective factor scores and for the subscales. Because of the lack of random assignment to preschool groups (parents selecting which type of preschool class for their children’s enrollment), an analysis of variance test was conducted to determine whether pretest means of the total protective factors scores differed significantly across the three groups (nature, blended, and traditional). Dependent *t*-tests were conducted to determine whether each group had significant growth in total protective factors.

To compare growth in protective factors of nature preschoolers to preschoolers attending blended and traditional classes, general linear modeling was used to investigate whether post-test levels of the protective factors (total score and subscales) significantly differed across groups, when controlling for pretest levels, as well as gender and dosage. In the models, the type of preschool (nature, blended, or traditional) served as the independent variable, and the dependent variable was the within-child protective factor, as measured by the DECA-P2 (initiative, self-regulation, attachment, and the combined total protective factors measure). Pretest scores, gender, and dosage of participation were covariates in the models. Age was not a covariate, per LeBuffe and Naglieri (2012) indicating protective factors do not vary much across the three- to five-year-old developmental period (initial models were run, however to check this; results confirm the decision not to include age into the analyses as a covariate). Nor was socio-economic status, ethnicity or race a covariate in the analyses, due to the lack of variation among participants. For significant models, pairwise comparisons were used to determine which groups had significant differences in adjusted post-test means between them.

In addition to the models where the independent variable was the preschool categorization (nature, blended, and traditional), general linear modeling was used with teacher as the independent variable (with six levels of this factor; these six teachers’ classes differed by their level of “nature-ness”). This was done in light of the rubric responses of teachers’ and their corresponding “nature-ness” scores, indicating more of a continuum of nature-ness rather than clearly discrete categories, and thus, the possibility that post-test scores differed by degree of nature-ness. It also provided a way to confirm the results from the models where preschool categories served as the independent variable to see whether differences across the groups “held up” or whether instead within-group variation across the teachers/classes was responsible for between-group differences.

## Results

Descriptive statistics for the preschool categories are reported in Table 3. Results of the comparison of pretest levels of total protective factors scores indicate there were no significant differences across nature, blended, and traditional classes, when controlling for gender, *F*(2)=0.99, *p*=0.37. This suggests that family-level nature engagement outside of preschool time is less of a concern in terms of interpreting the effects of nature preschool participation. If out-of-school time in nature, particularly by families who chose nature preschool, were influencing protective factors, there likely would have been significant differences across the pretest scores.

**Table 3:** Descriptive statistics for protective factors by category and teacher.

	Initiative		Self-regulation		Attachment/relationships		Total protective factors	
	Pretest M (SD)	Post-test M(SD)	Pretest M (SD)	Post-test M(SD)	Pretest M (SD)	Post-test M (SD)	Pretest M (SD)	Post-test M(SD)
Traditional (Combined Group, *n*=36)	54.16 (10.41)	55.42 (10.59)	51.68 (8.76)	54.33 (9.50)	49.95 (7.82)	53.75 (7.92)	52.27 (9.35)	55.06 (8.33)
Tchr 1, Classes a,b	45.18 (5.43)	46.53 (6.78)	46.44 (6.90)	47.20 (7.20)	45.43 (5.92)	55.40 (5.34)	44.93 (5.73)	49.60 (5.14)
Tchr 2, Class a	57.28 (6.16)	56.85 (5.08)	53.29 (3.59)	56.71 (2.92)	47.29 (4.82)	45.57 (3.78)	53.43 (5.16)	53.71 (3.15)
Tchr 3, Classes a,b	62.85 (7.92)	64.21 (8.08)	56.85 (9.36)	60.79 (8.74)	56.43 (6.65)	56.07 (9.29)	60.07 (7.72)	61.57 (8.55)
Blended (Combined Group, *n*=20)	43.14 (9.38)	49.45 (12.68)	46.82 (11.44)	52.45 (11.29)	38.36 (9.42)	52.05 (12.03)	41.91 (9.54)	51.50 (12.03)
Tchr 4, Classes abc	43.14 (9.38)	49.45 (12.68)	46.82 (11.44)	52.45 (11.29)	38.36 (9.42)	52.05 (12.03)	41.91 (9.54)	51.50 (12.03)
Nature (Combined Group, n=25)	47.46 (6.09)	61.52 (8.16)	50.17 (7.15)	57.16 (8.80)	53.53 (7.54)	61.52 (9.70)	50.64 (6.70)	61.44 (8.36)
Tchr 5, Classes a,b	48.60 (7.22)	58.92 (8.16)	48.73 (7.00)	57.00 (8.78)	49.40 (6.95)	55.23 (8.96)	48.80 (7.62)	58.15 (9.37)
Tchr 6, Class a	46.15 (4.39)	64.33 (7.50)	51.85 (7.25)	57.33 (9.22)	58.30 (5.08)	68.33 (4.49)	52.77 (4.90)	65.00 (5.50)

Based on the results of the dependent *t*-tests, the total protective factors of nature preschoolers increased over the course of the school year, and this growth was significant, t(24)=7.68, *p*<0.001. Preschoolers in the blended and traditional classes also had significant growth in protective factors, t(19)=5.66, p<0.001 and t(35)=3.65, *p*=0.001, respectively. The adjusted post-test means (post-test means when controlling for the pretests scores, gender, and dosage of participation) for each of the dependent variables from the general linear modeling analyses are reported in Table 4 (for both the group-level and teacher-level modeling). Table 5 provides a summary of the significant differences in the adjusted post-test means of the protective factors by group and teacher.

**Table 4:** Summary of adjusted post-test means^a^ by group and teachers.

	Initiative Estimated Mean (Standard Error)	Self-Regulation Estimated Mean (Standard Error)	Attachment/Relationships Estimated Mean (Standard Error)	Total Protective Factors Estimated Mean (Standard Error)
Traditional	51.41 (1.23)	52.97 (0.98)	52.71 (1.35)	52.65 (1.02)
Tchr 1, Classes a,b	50.50 (1.85)	50.12 (1.50)	57.43 (1.91)	53.56 (1.61)
Tchr 2, Class a	50.16 (2.73)	54.42 (2.14)	46.33 (2.73)	50.36 (2.27)
Tchr 3, Class b	52.92 (2.36)	55.62 (1.59)	50.82 (2.14)	52.69 (1.88)
Blended	55.60 (1.71)	54.79 (1.37)	58.31 (2.12)	57.42 (1.49)
Tchr 4, Classes a,b,c	55.60 (1.71)	54.79 (1.37)	58.31 (2.12)	57.42 (1.49)
Nature	62.37 (1.49)	57.25 (1.23)	58.02 (1.78)	60.12 (1.26)
Tchr 5, Classes a,b	59.44 (1.88)	58.21 (1.55)	54.45 (2.10)	58.49 (1.63)
Tchr 6, Class a	67.15 (1.98)	56.02 (1.62)	61.79 (2.38)	62.14 (1.73)

a *Controlling for Pretest Mean, Gender, and Dosage of Participation*.

**Table 5:** Summary of significant differences in the adjusted post-test means of the protective factors by group and teacher.

	Nature v. Traditional		Nature v. Blended		Blended v. Traditional	
	Evidence of Significant Difference from Comparisons of Categories	Evidence of Significant Difference from Comparisons of Classes of Teachers	Evidence of Significant Difference from Comparisons of Categories	Evidence of Significant Difference from Comparisons of Classes of Teachers	Evidence of Significant Difference from Comparisons of Categories	Evidence of Significant Difference from Comparisons of Classes of Teachers
Initiative	Yes	Yes	Yes	Yes	–	–
Self-Reg	Yes	Yes	–	–	–	Yes
Attachment	Yes	Yes	–	–	Yes	Yes
Total Protective Factors	Yes	Yes	–	–	Yes	Yes

### Initiative

Regarding the protective factor of initiative, there was a significant difference across preschool categories, F(2)=15.22, p<0.001, which corresponded to a large effect size (partial eta squared=0.29). The pairwise comparisons indicated significant differences between the nature and traditional preschool categories (mean difference 11.00, SE=2.00, p<0.001) and between the nature and blended categories (mean difference=6.77, SE 2.33, *p*=0.01). The difference in adjusted initiative post-test means between the nature-lite (blended) and traditional categories was not significant (Mean Difference=5.59, SE=2.19, *p*>0.05).

When the analysis was run with teacher as the independent variable, there was a significant difference across teachers, *F*(5)=10.58, p<0.001, which corresponded to a large effect size (partial eta squared=0.42). The class of teacher six (categorized by the district as nature; nature-ness score of 36.5) had a significantly higher adjusted post-test mean than all of the other classes (p<0.001). The two combined classes of teacher five, also categorized by the district as nature and a nature-ness score of 36.5, had a significantly higher adjusted post-test mean than the classes of the three teachers in the traditional preschool category, with their nature-ness scores of 22, 29, and 29.5, respectively (*p*=0.001, *p*=0.01, *p*=0.04, respectively), but not significantly higher than the teacher’s classes that were classified as blended by the district and had nature-ness score of 33 (*p*>0.05). The three teachers’ classes in the traditional category did not significantly differ among themselves (p>0.05) nor did the teacher’s combined classes that were categorized as blended differ from teachers’ classes in the traditional category (p>0.05).

### Self-Regulation

Regarding the protective factor of self-regulation, there was a significant difference across preschool categories, *F*(2)=3.65, *p*=0.03, which corresponded to a medium to large effect size (partial eta squared=0.09). The pairwise comparisons indicated significant differences between the nature and traditional preschool categories (mean difference 4.28, SE=1.60, p=0.01). The difference in adjusted self-regulation post-test means between the nature and blended categories was not significant (mean difference=2.18, SE=1.75, p>0.05) nor was it significant between the blended and traditional categories (Mean Difference=1.92, SE=1.64, p>0.05).

When the analysis was run with teacher as the independent variable, there was a significant difference across teachers, *F*(5)=3.06, *p*=0.02, which corresponded to a large effect size (partial eta squared=0.17). The classes of teachers six and five (which were categorized by the district as nature; nature-ness scores of 36.5) had a significantly higher adjusted post-test mean than the classes of teacher one, who was classified as traditional by the district and a nature-ness score of 22 (*p*<0.001, p=0.01, respectively). The adjusted post-test means of the combined classes of teacher four (categorized as blended, with a nature-ness score of 33) and the combined classes of teacher three (categorized as traditional, with a nature-ness score of 29.5) were also both significantly higher than teacher one (*p*=0.02 for both).

### Attachment

Regarding the protective factor of attachment, there was a significant difference across preschool categories, F(2)=4.46 *p*=0.02, which corresponded to a medium to large effect size (partial eta squared=0.11). The pairwise comparisons indicated significant differences between the nature and traditional preschool categories (mean difference 5.31, SE=2.21, p=0.02) and between the blended and traditional categories (mean difference=5.56, SE 2.56, *p*=0.03). There was not a significant difference between the nature and blended categories (mean difference=0.23, SE=2.90, *p*=0.94).

When the analysis was run with teacher as the independent variable, there was a significant difference across teachers, F(5)=6.06, *p*<0.001, which corresponded to a large effect size (partial eta squared=0.29). The classes of teachers five and six (categorized by the district as nature; nature-ness scores of 36.5) were significantly higher than the class of teacher two (categorized as traditional; a nature-score of 29), p=0.02 and *p*<0.001, respectively, and the combined classes of teacher four (categorized as blended; nature-ness score of 33) were also higher than the class of teacher two, *p*=0.001. The class of teacher six and the combined classes of teacher four were both significantly higher than the combined classes of teacher three (categorized by the district as traditional with a nature-ness score of 29.5), *p*=0.02 and *p*<0.001, respectively. There was also significant within-category variation. The combined classes of teacher one (traditional; nature-ness score of 22) were significantly higher than the classes of teachers two and three (traditional; nature-ness scores of 29 and 29.5), p=0.001 and p=0.03, respectively. The combined classes of teacher five (nature) were significantly higher than the class of teacher six (nature), p=0.02; both teachers had nature-ness scores of 36.5.

### Total Protective Factors

When the subscales (individual factors of initiative, self-regulation, and attachment) are combined into the measure of total protective factors, there was a significant difference across preschool categories, *F*(2)=11.25 p<0.001, which corresponded to a large effect size (partial eta squared=0.23). The pairwise comparisons indicated significant differences between the nature and the traditional preschool categories (mean difference 7.46, SE=1.63, *p*<0.001) and between the blended and traditional preschool categories (mean difference 4.82, SE=1. 85, *p*=0.01). There was not a significant difference between nature and blended (mean difference 2.92, SE=1.91, *p*=0.13).

When the analysis was run with teacher as the independent variable, there was a significant difference across teachers, *F*(5)=5.87, *p*<0.001, which corresponded to a large effect size (partial eta squared=0.29). The classes of teachers five and six (categorized by the district as nature; nature-ness score of 36.5) were significantly higher than the classes of teachers three, two, and one (categorized by the district as traditional; nature-ness scores of 29.5, 29, and 22, respectively), *p*=0.02 for teacher five across the comparisons with the traditional classes, and *p*<0.001 for teacher six across the comparisons with the traditional classes. The combined classes of teacher four (categorized by the district as blended; nature-ness score of 33) were significantly higher than the class of teacher two (categorized as traditional; nature-ness score of 29), *p*=0.02.

## Discussion

### Limitations

It is important to consider these findings within the context of the study’s threats to validity. In light of lack of random assignment limiting internal validity, it is difficult to attribute results solely to participation in the type of preschool. While pretest scores were incorporated into the analyses to account for possible pre-existing differences and despite participants being from within the same school district, cautious interpretation and generalization is warranted. Also, while the DECA user manual was followed regarding guidance regarding age and gender in the statistical modeling, it is important to note that the sample from this study was different from the national sampling and analyses conducted by the test authors toward the normed data and recommendations regarding use of covariates. Another limitation stems from the “nesting” of data (children within classes within teachers within categories). The analysis approach used was selected in place of multi-level modeling because of an insufficient sample size at the program level and due to the groups being a fixed rather than random factor, per recommendations by Garson (2013) and Huta (2014). However, there is the possibility of inaccurate statistical estimates from not accounting for the hierarchical structure of the data and the resulting risk of partitioning variance incorrectly (Woltman et al., 2012).

Construct validity is limited due to the single measure of total protective factors associated with resilience and also due to the potential for hypothesis guessing, particularly when teachers were associated with both the independent and dependent variables. Construct validity is also limited from mono-operation bias, as there were not multiple classes for two of the teachers. Additionally, these two teachers had fewer students overall for both observing students and completing the research instrument, further threatening construct validity. Similarly, there was only one teacher for the three classes in the blended category and thus only one “rater” completing the DECA instrument. Further, the self-report nature of the measure of teachers’ levels of “nature-ness” is another threat to the construct validity of the study. In addition, teachers may have varied in their level of childhood teaching experience, degree and licensure/emphasis, and experience with nature-based practices; these may have impacted not only how their curriculum and instruction, but also how they completed the DECA research instrument, particularly since there is no training for using the DECA.

External validity is limited given the voluntary participation and also the lack of variation in terms of race, ethnicity, and socio-economic status of the sample at hand. Additionally, it is important to restate this study was conducted during a pandemic, which further limits the external validity of the study. While pretest levels of participants’ protective factors were within the normative range reported in the DECA manual, the conditions children experienced throughout the school year likely negate comparisons with published test norms and perhaps limit the external validity of this study beyond pandemic times. It is unclear from the findings at hand whether the growth in total protective factors among the nature preschool participants was further influenced by children having transitioned out of a time period in which they were primarily homebound with potentially elevated stress levels within households. Thus, the immersion in nature and the opportunities for unstructured outdoor play may have been even more salient, thereby strengthening the efficacy of nature preschools beyond what might occur during non-pandemic times. These limitations, individually and collectively, are important to consider when drawing implications from the study’s findings.

### Discussion of Findings

These results overall suggest that nature and blended preschool classes were effective in supporting growth in total protective factors. Thus, when goals for young children include fostering the protective factors children can draw upon in times of adversity, the incorporation of nature-based practices and experiences into preschool programming appears to be an effective approach. Nature preschools seem particularly effective, as children’s protective factors at the end of the preschool year corresponded with the descriptor, “strength,” whereas the preschoolers in the blended and traditional sections had protective factors at the level of “typical” for their age, per guidelines in the DECA User’s Guide and Technical Manual (LeBuffe and Naglieri, 2012). However, in light of some variations in the results pertaining to the protective factors individually, it may be useful to consider the factors individually toward guiding practice and further research.

Regarding initiative, results suggest it is being furthered through nature preschool, more so than traditional preschool, and likely more so than blended preschool. In other words, the degree of nature-ness of the participating teachers/classes seemed to impact initiative, and if the goal is increasing or maximizing the protective factor of initiative in young children, nature preschool appeared be most effective. Blended preschool in this study seemed to be no more effective than traditional preschool in terms of supporting initiative in preschool-aged children.

Regarding self-regulation, results suggest it was supported through nature preschool, more so than through traditional preschool. Additionally, the degree of nature-ness in the participating teachers/classes appeared to influence the effectiveness on self-regulation, with a greater degree of nature-ness being more effective than a lesser degree of nature-ness, particularly when comparing blended and traditional classes.

Regarding attachment/relationships, results suggest both that nature and blended preschool classes were more effective in supporting it in young children than traditional classes. In light of the within-category variation in attachment levels, there was likely some other teacher and/or programming characteristic that was influencing attachment, other than the degree of nature-ness; whether this other characteristic was as influential as nature-ness is unknown.

These results also suggest the greatest impact of nature preschool on initiative, as this individual protective factor had the greatest effect size relative to the others. This also was the only protective factor with evidence suggesting nature preschool was even more effective than blended preschool. One possible explanation could be attributed to the less-structured approach within the nature preschool category; the majority of the day is not teacher-directed, and there is a substantial focus on unstructured, child-directed nature play. Thus, students have more autonomy and free choice to choose the activities they want to take part in, and to participate (regardless of what that looks like or entails) takes initiative. The natural spaces for children to explore in nature often lead to less supervision and increased distance from teachers, which affords opportunities to problem solve on their own, rather than relying on a teacher for help (Alme and Reime, 2021). Grey (2013) positions increasing anxiety and declining resilience as resulting from the dramatic decline in children’s opportunities to playfully explore and pursue their own interests away from adults. In nature preschool, children are more responsible for coming up with ideas regarding what to play, for solving problems when they arise, assisting each other as they encounter and initiate challenging activities, rather than relying on teachers for things they can do for themselves. Additionally, the dynamic nature of natural outdoor settings continuously affords opportunities for children to constantly adapt and problem solve, which prompt the opportunity for initiative (Alme and Reime, 2021).

For self-regulation, attachment, and the combined measure of total protective factors, results suggest that some incorporation of nature experiences and practices is better than none and that nature preschool may not lead to even stronger outcomes than blended approaches. In a study by Kochanowski and Carr (2014), child-directed nature play was associated with an increase in self-regulation. Their study suggested nature’s open-ended structures and loose parts challenged children’s physical boundaries; consequently, children often displayed their courage and determination through continued attempts to succeed. The study authors speculate that through these experiences, children often experienced a mix of emotions including frustration and anger, and by continuing to not give up, students exercised and developed skills related to self-regulation. Perhaps, since open-endedness and loose parts were features of play in both nature playscapes and unmaintained natural settings and since children in the blended preschool classes had the opportunity to play in playscapes, it is reasonable to expect some growth in self-regulation for both blended and nature sections. This illustrates that depending on the desired outcome at hand, the dosage of “nature” (whether that be in terms of setting, time, or time proportional to another type of activity) likely matters.

The possibility that incorporating some nature-based practices can be influential, whether that be on self-regulation and attachment or other outcomes, is noteworthy. Not all preschools can or want to become nature preschools. An incremental shift for programs might make more sense for programs wanting to experiment with the feasibility and impact of integrating nature-based experiences and settings into their programming. Also important to note, though, is that it is unlikely that self-regulation or attachment, nor even protective factors associated with resilience overall, would be the sole aim for a preschool program. Thus, while blended approaches (incorporating some nature-based approaches and settings) may suffice for fostering attachment or self-regulation, there are likely other important developmental outcomes that perhaps may be impacted less so without the full degree of nature-ness in the program. This study suggests initiative is one of those outcomes.

What does this mean for policy makers and funders? The study at hand is encouraging, as it suggests that for relatively little investment, meaningful and timely impacts (strengthening of protective factors relating to resilience) might be gained. For example, in this study, children playing on a shrub/vegetation-covered slope with access to loose parts appear to have had increases in self-regulation and attachment over the course of the school year. While this unstructured play was daily, it was not for unreasonably lengthy periods of time (about an hour a day). However, the nature play was consistent; it was not dependent on weather or seasons. Thus, perhaps rather than large financial investments, funding organizations could encourage this type of play through small grants to support small-scale projects to naturalize school grounds, or through other means, such as helping preschools identify places on their school grounds where outdoor play with natural loose parts could happen. Or perhaps the investment comes in the form of outdoor clothing or footwear that makes outdoor play more feasible in a range of weather conditions and seasons. Another investment, for example, may be along the lines of fostering among preschool teachers the receptivity, motivation, and commitment toward daily outdoor play with natural elements as well as skills for navigating barriers that arise (perhaps through networks or mentors who can help “troubleshoot” challenges that arrive). Or perhaps the investment comes in the form of early learning and care policies that encourage rather than discourage outdoor play in nature. At the same time, it is important to be both mindful of the range of relevant early childhood learning and developmental outcomes and intentional in action, investing in strategies, materials, and settings that match the desired outcome at hand.

### Implications for Further Research

Due to the pandemic and the University’s restrictions on face-to-face data collection, observation data were not collected. Nor were parents asked to complete the DECA-P2, to avoid adding further stress in the midst of the uncertainties surrounding the upcoming school year. Future research might entail incorporating multiple sources of data such as these toward a more complete understanding of the impact of nature preschool on total protective factors. Also in light of the study being conducted during the pandemic, future research exploring the impact of nature preschool on total protective factors during non-pandemic times would lend insight into a potential association between nature preschools’ efficacy and the presence of adverse conditions. With growing evidence of risks to health and well-being posed by adverse life experiences that occur during critical developmental periods, particularly when adversities are prolonged or cumulative (Masten and Barnes, 2018), this research direction could have significant implications for practice, particularly if an association were found.

Additionally, while these results show a positive relationship between nature preschool participation and fostering protective factors related to psychological resilience, there are areas where further research is necessary. For example, within the subscale of attachment, results suggest there is likely an equally strong or stronger influence on attachment other than degree of nature-ness, particularly with the higher levels of attachment within teacher one’s traditional preschool classes. A possible explanation could be that the role of the adult/teacher in a traditional classroom is more hands-on and teacher-directed, whereas in nature sections, there is much child-directed free play and potentially less interaction with teachers. Showing preference for and seeking help from an adult are indicators of attachment, and therefore, differences in the degree of teacher-directed interactions may provide at least a partial explanation. Also, attachment seems potentially most likely to have been affected by quarantine and distance learning due to COVID-19. Thus, more research is needed to explore not only attachment, but more generally, investigating what about nature preschools has a positive influence on protective factors, individually and collectively, and investigating the durability of these gains beyond the preschool year.

Chawla et al. (2014) and Ernst et al. (2018) speculate as to what about nature preschool may prompt these positive findings, yet given the importance of resilience, research that allows for more than speculation on the mechanisms is critical toward guiding practice (both teacher professional development and nature preschool implementation). The design of this study limits the ability to attribute the positive impact to any particular program characteristic; nor is it clear from this study whether nature preschools are responsible for the increase in protective factors or if instead, for example, they are an effective vehicle for providing time for children to be in nature, with time in nature being the source of positive impact. Since pretest levels of protective factors did not significantly differ across the groups, it would reason that family nature engagement and/or time in nature is not solely responsible for the findings at hand. Further research, though, is needed to better understand which program characteristics are most influential and how program characteristics interact to support the development of protective factors (e.g., is it the frequent and sustained periods of time in nature, or is it the unstructured play and child-directed interactions, or is it the interactions with preschool peers afforded by nature play?). Future studies might incorporate additional comparison groups, such as child-directed, play-based preschool programs (e.g., Montessori preschool programs) or “drop-in” nature play programs for parents and preschool-aged children that do not have the structure and format of a nature preschool. Future studies might also benefit from including the amount of time children spend in nature outside of the preschool day as a covariate in the analyses.

Another direction for further research relates to investigating whether the effectiveness of nature preschool on protective factors varies based on race, ethnicity, and socio-economic status. This would be helpful toward establishing external validity of the study. Furthermore, research has found that economic hardship severely and adversely impacts child development, learning, and quality of life and that not all races experience adverse childhood experiences equally (Sacks and Murphey, 2018). While black non-Hispanic children experience the most occurrences of adverse childhood experiences (Sacks and Murphey, 2018), they are among the least represented within nature preschools [North American Association for Environmental Education (NAAEE), 2017]. Understanding the effectiveness of nature preschools on children’s protective factors across races and ethnicities could have urgent implications for diversity, equity, and inclusion-related concerns within the nature preschool movement, particularly if it is determined that nature and/or blended preschools are effective or even more effective for races and ethnicities currently underrepresented in the current nature preschool movement. As such, the potential exists for nature preschool to further educational and developmental disparities, especially when lack of research exists on possible treatment by demographic interaction effects and in light of lack of representativeness within the nature preschool movement.

## Conclusion

This research sought to examine the impact of nature preschool on the growth of protective factors associated with resilience and the impact relative to that in blended and traditional preschool classrooms. Given the prevalence of adverse childhood experiences, the pandemic that children have just experienced, and the range of day-to-day adversities encountered in life, resilience is a relevant and significant construct to support within young children. The results of this study suggest that when we invest in nature-based early learning and integrate child-directed nature play into the preschool day, the returns are not only significant growth in total protective factors, but protective factors that are above typical for this age level and at a level corresponding with being considered “strengths.” Further, this study’s findings suggest that we can maximize the return on investment, particularly in the case of the protective factor of initiative, through nature preschools, yet for furthering self-regulation and attachment, some incorporation of nature-based practices may also be effective. Considering these results alongside existing literature, this study adds to the evidence base supporting the use of nature-based practices and settings for supporting children’s well-being and opens the door for encouraging programs to incorporate even some aspects of nature-based practices toward helping children develop the skills for navigating the challenges that may lie ahead. However, as this study was exploratory, future research is needed to confirm associations, as well as to untangle moderating and mediating factors, toward identifying which elements of nature preschool need to be studied further in order to more precisely articulating the return associated with nature preschools.

## Data Availability Statement

The datasets presented in this article are not readily available because this data is regarding young children, and parents were not asked to consent to inclusion of their children’s data in a dataset that may be shared with other researchers. Requests to access the datasets should be directed to jernst@d.umn.edu.

## Ethics Statement

This study involved human participants and was reviewed by the University of Minnesota Institutional Review Board. Written informed consent to participate in this study was provided by the participants’ parent or legal guardian.

## Author Contributions

DS and JE were the principal investigators and designed and provided oversight for the study. HJ was the graduate student research assistant and responsible for the literature review, scoring of the assessments, and data entry; this study was completed as her master’s thesis. JE was responsible for data analysis and led the manuscript preparation. DS and HJ contributed to the manuscript writing and review. All authors contributed to the article and approved the submitted version.

## Funding

The George B. Storer Foundation provided funding for this study.

## Conflict of Interest

The authors declare that the research was conducted in the absence of any commercial or financial relationships that could be construed as a potential conflict of interest.

## Publisher’s Note

All claims expressed in this article are solely those of the authors and do not necessarily represent those of their affiliated organizations, or those of the publisher, the editors and the reviewers. Any product that may be evaluated in this article, or claim that may be made by its manufacturer, is not guaranteed or endorsed by the publisher.
